# Income distribution trends and future food demand

**DOI:** 10.1098/rstb.2010.0164

**Published:** 2010-09-27

**Authors:** Xavier Cirera, Edoardo Masset

**Affiliations:** Research Fellows, Institute of Development Studies, University of Sussex, Brighton, UK

**Keywords:** income distribution, food demand, Engel's law

## Abstract

This paper surveys the theoretical literature on the relationship between income distribution and food demand, and identifies main gaps of current food modelling techniques that affect the accuracy of food demand projections. At the heart of the relationship between income distribution and food demand is Engel's law. Engel's law establishes that as income increases, households' demand for food increases less than proportionally. A consequence of this law is that the particular shape of the distribution of income across individuals and countries affects the rate of growth of food demand. Our review of the literature suggests that existing models of food demand fail to incorporate the required Engel flexibility when (i) aggregating different food budget shares among households; and (ii) changing budget shares as income grows. We perform simple simulations to predict growth in food demand under alternative income distribution scenarios taking into account nonlinearity of food demand. Results suggest that (i) distributional effects are to be expected from changes in between-countries inequality, rather than within-country inequality; and (ii) simulations of an optimistic and a pessimistic scenario of income inequality suggest that world food demand in 2050 would be 2.7 per cent higher and 5.4 per cent lower than distributional-neutral growth, respectively.

## 1. Introduction

This paper focuses on the impact of income distribution on food demand, an issue that has been significantly overlooked by a large proportion of studies projecting future food demand. Specifically, the objective of the paper is to survey the theoretical literature in this area, and at the same time identify the main existing gaps in order to build more realistic food demand scenarios. To our knowledge, this is the first survey to focus on this area.

The ability to feed the world population in the near future depends critically on the capacity of food supply to meet an increasing demand. As population rises, more people need to be fed, and as income grows more households' disposable income is available for food consumption. While there is little doubt that the demand scenario for the next decades is one of positive growth, a crucial question is at what rate world food demand is expected to increase. This is particularly critical given the recent additional pressures on the food system arising from the increasing link between food and energy markets via biofuels.

One particular element that is essential for projecting food demand growth is income distribution. One of the most robust and stronger relationships in economics is ‘Engel's law’, after Ernst Engel, a German economist, who in the nineteenth century studied food consumption of the Belgian working class. This law establishes that as income increases, households' demand for food increases less than proportionally. Hence, as households become richer, their share of expenditure on food decreases until reaching a ‘saturation’ point, after which food demand is hardly responsive to any income increases.^1^

An important implication of Engel's law is, therefore, that income distribution changes are relevant when it comes to predicting future food demand. Concretely, the rate of growth of food demand over the next decades should be dependent on the way in which income growth will be distributed among households and countries. Faster income growth among poorer countries and households should result in faster food demand growth in the short and medium term, since poorer households and countries tend to allocate larger shares of their budgets for food consumption. However, if this more equalitarian growth scenario persists, we should expect faster reductions in food demand growth as poor countries converge more rapidly to the threshold of food ‘saturation’. On the other hand, more unequal growth scenarios, with slow income growth in less developed countries (LDCs), would imply slower demand growth in the short and medium term, but sustained in the long term, probably exacerbated by larger population growth in poorer countries.

The paper is structured as follows. Section 2 explores the theoretical channels through which income growth impacts food demand. Section 3 reviews the empirical evidence regarding estimation and simulation of Engel curves, and enumerates the desirable properties of demand systems in order to account for income distribution dynamics. Section 4 reviews and assesses models to forecast future food demand and the main projection assumptions used. Section 5 highlights with a simple illustrative example, the potential risks associated when income distribution dynamics are not included in demand forecasts. The final section concludes.

## 2. Income distribution and food demand: the channels

### (a) What is food demand?

For the purpose of this review, food demand is household consumption of food that is either purchased in the market or home-produced. Two facts should be kept in mind before starting the analysis. First, no household, however poor, consumes only food. There are a number of essential items like shelter and medications that even the poorest households buy. Second, food purchases are rarely dictated by nutritional requirements and people do not normally buy the food that is recommended by health providers as healthier or more nutritious. Food demand is dominated by tastes, which vary across countries and over time.

Food consumption can be disaggregated into categories in order to conduct more detailed analysis of changes in consumption patterns. The standard procedure for the aggregation of food items consists of grouping together items that are close substitutes in consumption. For example, tea and coffee are often grouped in the ‘stimulants’ category. Similarly, pork, lamb, beef and chicken are grouped in a single ‘meat’ category. Table 1 below, shows the expenditure shares of seven food categories for a sample of rural Indian households, and is an example of such disaggregation. Notice how the small share of food expenditure on meat is a reflection of both tastes and poverty of this sample of households.

**Table 1.** Food expenditure shares in rural India (2004–2005). Source: calculated from NSSO–National Sample Survey Office (2006)–India data.

food category	share
cereals	38.2
pulses	6.4
dairy products	9.9
oils and fats	9.1
meat, egg and fish	7.7
vegetable and fruit	14.8
other	13.9

### (b) Income and food demand. Engel's law and the problem of aggregation

As income increases, households' demand for goods, including food, increases. It has been documented by innumerable empirical studies that food demand increases less than proportionally with income. This relationship between food consumption and income is described by Engel curves (Engel's law).

The nonlinearity of the food Engel curve has a strong implication on the relationship between income and food demand; food is not only a function of income, but also of the income distribution within the economy. To illustrate this, consider the following linear food demand of a consumer, where *q* is food demand, *a* can be interpreted as a minimum consumption level of food, *y* is income and the observations are indexed across households or individuals (i):
2.1
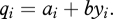



Adding up all the minimum individual consumption levels (*a*_*i*_) and incomes (*y*_*i*_) over all households, we obtain total food consumption (*Q*) as a linear function of total income in the economy (*Y*)
2.2
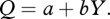



The problem with this formulation is that it is not valid if food Engel curves are nonlinear. Suppose, for example, that we transfer income from a rich household to a poorer one. According to equation (2.2), food demand in the economy does not change because total income has not changed. However, we know this is not true. Because Engel curves are nonlinear, the poor tend to spend more on food than the rich, and an income transfer from the rich to the poor, while preserving income constant, will increase food demand.

**Figure 1.**
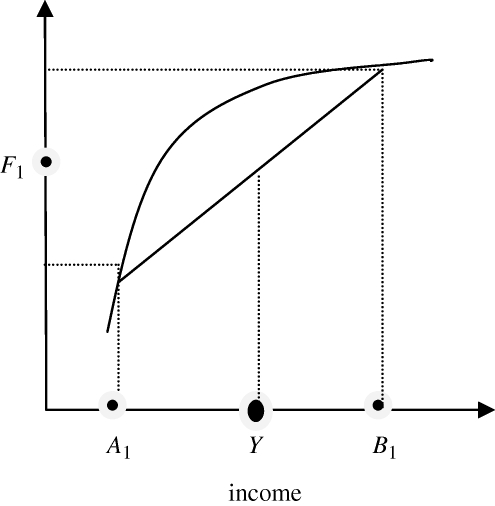
Average food demand in a two-individual economy.

Some graphical analysis may help to further illustrate this point. Suppose the economy is composed of only two individuals, one (*B*) is rich while the other (*A*) is poor. Their average income is *Y*, while their average food consumption given the shape of the Engel curve is *F*_1_ (figure 1).

**Figure 2.**
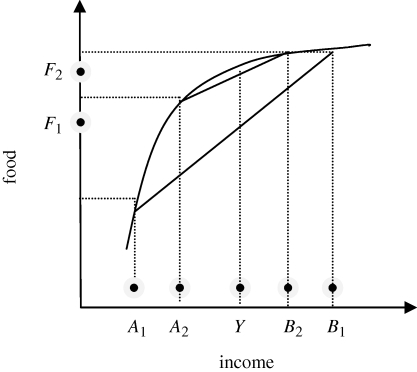
Effect of an income transfer on food demand in a two-individual economy.

Consider now a more equitable income distribution between individuals *A* and *B*. Some income is taken from the rich (*B*), and given to the poor (*A*). While average income *Y* remains unchanged, average food consumption has now increased to *F*_2_ (figure 2). After the transfer, food consumption of the rich has only marginally decreased, while food consumption of the poor has increased dramatically.

This brief discussion helps in emphasizing two main points. First, the aggregate demand for food is not only a function of income but also of income distribution. Using the variance in the distribution of income (*σ*_*y*_) as an index of inequality, equation (2.2) can be re-written in the following way:
2.3
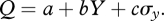



Furthermore, an increase in inequality has the effect of reducing aggregate food demand, while a decrease in inequality has the effect of increasing aggregate food demand.

The difficulty in aggregating individual households consumption behaviours described above is known in demand theory as *the aggregation problem*: the transition from micro- to macroeconomics of consumer behaviour (Deaton & Muellbauer 1980). The literature on aggregation dates back at least to Antonelli (1886) and has shown that exact aggregation of individual consumer demands is possible only if Engel curves are linear (Blundell & Stoker 2005), which is very unlikely to be the case for most products and certainly not for food. In addition, changes in the demographic structure of the household over time also have implications for aggregation and changes in shape of the Engel curve over time.^2^

### (c) Cross-substitution and other commodities: Bennet's law

As income increases, not only does the quantity of food increase less than proportionally, but the composition of the food basket also changes. In particular, it has been observed that the consumption of starchy staple food declines with income. This fact has been labelled ‘Bennet's law’, after Bennet, who was the first to document the decrease in the amount of calories that people obtain from starchy staples versus other food as income increases (Timmer *et al*. 1983).

**Figure 3.**
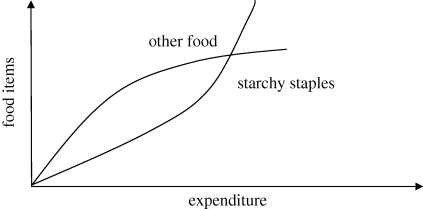
An illustration of Bennet's law.

Food is an aggregate of a large number of consumption good. For example, the food expenditure questionnaire used by the National Sample Survey Organization (NSSO) of the Government of India (2006) collects information on nearly 150 food items. Clearly, while in the aggregate the consumption of food increases less than proportionally with income, the consumption of some specific items may increase. These items, whose consumption increases more than proportionally with income, are often called ‘luxuries’ in the economic literature in contraposition to ‘necessities’. Bennet's law is portrayed in figure 3. While the consumption of starchy staples increases less then proportionally with food expenditure, the aggregate consumption of other food item must increase.

The effect of a change on the distribution of income on consumption of a particular good depends on the shape of the Engel curve. In addition to the concave down form already analysed, two other forms are possible: linear and concave up. A linear Engel curve for a good is distribution-neutral. Changes in the distribution of income, while preserving the existing level of income, have no effect on the consumption of that good. In effect, the linearity of the Engel curve was found to be the condition that allows the exact aggregation of single Engel curves across consumers. If, on the other hand, the Engel curve is concave up, an increase in inequality increases consumption of that good. For example, Engel curves for health expenditure are often exponential, meaning that an increase in income inequality raises aggregate expenditure on health.

Few food items display concave up Engel curves. For example, food categories such as ‘beverages and tobacco’, ‘fish’ and ‘food outside home’ may have expenditure elasticities above one (e.g. Seale *et al*. 2003). More typically, Engel curves for food categories are linear or bending downwards. In the latter case, consumption increases proportionally with income for the poor and the middle classes, but then falls again for the rich. Consumption of meat and fats in particular tend to have an inverted U shape of this type. In this latter case, it is difficult to predict the consumption effect of changes in income distribution. In order for this to be calculated, the shape of the Engel curve, and the form of the change in the distribution of income, need to be known by income quintile.

### (d) Dynamic analysis and the transition to food ‘saturation’

The aggregation problem described above refers mainly to the capacity to represent all households' demand at a point in time. However, when thinking about the impact of income distribution on food demand in the long term, one needs to consider how households and countries move along the Engel curve. Historically, diets in industrialized countries have shifted from traditional grains towards meat, dairy products and protein-based food, and changes towards more balanced and healthy diet has also reduced income demand elasticities reaching the level of almost ‘saturation’. Similarly, reductions in food budget shares have been observed in other regions and countries.

When dynamic effects are considered, the impact on food demand may result in significantly different outcomes in the short, medium and long term. Paradoxically, in the short and medium term, a scenario of pro-poor growth that reduces inequality by a faster increase in *per capita* income in developing countries is translated into higher demand growth, since income grows more for those households with higher food demand elasticities. However, in the long term, if equalizing growth is persistent, developing countries will hit the ‘saturation’ point faster, implying higher demand growth but at decreasing rates. This is particularly important for very populated countries such as China, where successes in lifting people out of poverty and increasing the middle class will be translated into a deceleration on food demand growth.

## 3. The empirics of engel curves

### (a) Estimation and simulation of food Engel curves

A major concern when estimating and modelling demand systems is the need for some degree of Engel flexibility. Often demand systems have been used in empirical and modelling studies based on goodness of fit or tractability, rather than consistency with observed consumer behaviour. Specifically, consistent demand systems should have the following properties:

— Ability to represent luxuries (commodities whose consumption increases more than proportionally with income), necessities (whose consumption increases less than proportionally with income) and inferior goods (commodities whose consumption decrease as income increases).
— They allow the same commodity to be a luxury for the poor but a necessity for the rich.
— They do not violate the adding-up condition that consumers do not spend more than their income, and budget shares are below unity.

More flexible demand systems have been developed in recent years using semi-logarithmic forms and different polynomials on the expenditures terms.^3^ These more flexible forms have crystallized in the development of rank three demand systems (Lewbel 1991). The rank of a demand system is the maximum dimension of the function space contained by the Engel curve. In practical terms, rank one demand systems generate constant elasticities independent of income/expenditure. Rank two systems generate linear Engel curves, while rank three produce nonlinear Engel curves.

Although still not widely implemented, these more Engel flexible demand systems, such as an implicit additive demand system (AIDADS) and the quadratic almost ideal demand system (QUAIDS) (See the electronic supplementary material, appendix for a more detailed description of these systems), are starting to be used in modelling exercises. The integration of rank three demand systems in empirical and modelling work is essential if one wants to account for the observed dynamic effects of falling marginal food budget shares.

### (b) A representative consumer: how important are errors from aggregation?

While more flexible Engel demand systems correct unrealistic dynamic predictions that do not account for movements along the Engel curve, these systems do not correct the problem of aggregation described above. Most demand systems are based on a representative consumer. Therefore, if the bias of aggregation is significant, this would trigger further biases when estimating future food budget shares.

Several authors have analysed empirically the importance of the aggregation problem. Lewbel (1991) shows that aggregation biases are small under certain demand systems (i.e. PIGLOG) and forecasts based on a representative consumer will have low aggregation error if income distribution dynamics remain stable. Moreover, these differences can be minimized when considering the distribution of expenditures, although exact aggregation models will fit the data better. In addition, and more importantly, demographic changes, and changes in the age composition of the household, are potentially a larger source of aggregation bias than nonlinear income effects. Denton & Mountain (2001) show that aggregation errors are small when using an almost ideal demand system (AIDS) and assuming that income is distributed lognormal. Denton & Mountain (2007) estimate the aggregation biases under different income distribution configurations using the AIDS system and its quadratic version (QUAIDS). Overall, the authors find that differences are small, and only for a few configurations of large elasticities and high inequality the biases can become very significant.

The evidence suggests, therefore, that aggregation biases in the short term tend to be small under most demand systems. Nevertheless, the size of these biases depends on the stability of income distribution. As a result, biases can increase when performing longer term predictions, where departures from distribution-neutral income growth may be significant.

### (c) Partial or general equilibrium

Ray (1998) points out that changes in income distribution that generate changes in demand composition may in turn generate changes in factor demands. Changes in factor demands feed back into further changes in the distribution of incomes. For example, an increase in inequality may increase the demand for luxury goods, which in turn increases the demand for capital. If the production of the good whose demand has increased is capital-intensive, inequality will further increase. Theoretical models along these lines have been formulated by de Janvry & Sadoulet (1983), Bourguignon (1990) and Baland & Ray (1991).

**Figure 4.**
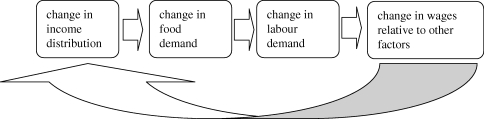
Changes in income distribution and food demand.

Figure 4 illustrates how the distribution of income has implications for the aggregate levels of food expenditure and vice versa. There is a feedback process via factors demand that affects the distribution of income. Depending on the values of the parameters of the general model, the initial shock in the income distribution may dampen or accelerate the initial change in food demand.

### (d) An optimal demand system to account for income distribution changes

In order for a demand system to represent food demand in a way that reflects changes in the distribution of income, within countries and across countries and over time, the following conditions must be met:

— Food demand elasticities need to vary within countries and between countries for different levels of *per capita* expenditure. Therefore, models need to produce Engel functions.
— Food demand elasticities need to vary over time as people and countries become richer. Rank three demand systems such as QUAIDS or AIDADS are able to generate these decreasing marginal budget shares.
— The demand system should minimize aggregation problems by using demands that aggregate individual or group demands, and estimate those as a function of the distribution of expenditure.^4^
— Feedback resulting from changes in factors' demand and factors' remunerations need to be incorporated in the model.
— Elasticities should be adjusted based on prediction of fertility rates and on the evolution of the demographic structure of populations.

In what follows, we briefly review to what extent existing models used to estimate and predict patterns of world food demand to meet the conditions outlined above.

## 4. Food demand models and forecasts

### (a) Projection assumptions

Even in the case of being able to build the ‘perfect’ model to forecast food demand embedded with the desired properties described above, the accuracy of the forecasts depend on the long-term assumptions regarding three key variables: population growth, income growth and income distribution. Before analysing existing models for predicting food demand, it is important to review the existing projections for these three variables.

#### (i) Population projections

The main source of population forecasts are those of the United Nations *World population prospects*. The projections indicate a slowdown of the world's population growth for all regions. World population is projected at around 9.1 billion in 2050, converging to growth rates per annum of around 0.3 per cent. This slowdown will be particularly important in China, hitting negative population growth from 2030 and driving negative population growth in East Asia. Developed countries will continue with very low growth, which is expected to turn negative around 2040. Population growth will slow down in South Asia and Sub-Saharan Africa, although this last region will experience larger population increases, around 1.3 per cent per annum.

This implies that higher population growth will occur in the regions with higher poverty and larger food demand elasticities and, therefore, income *per capita* growth in Sub-Saharan Africa will be key to determining the extent of food demand growth; although slower population growth will imply slower food demand growth.

#### (ii) Income growth projections

While population projections are more reliable owing to more persistent demographic dynamics, income growth dynamics are more uncertain. The main source for these projections is the World Bank *Global economic prospects* (2009). The projections are of substantial *per capita* income growth, although this is expected to be lower for all regions during the period 2015–2030 compared with the previous decade. This result is mainly driven by the fact that income in the large developing countries is expected to decelerate slightly. Despite this slowdown, *per capita* incomes in developing countries are expected to triple from $1550 to $4650 between 2004 and 2030 (World Bank 2009). Incidentally, World Bank (2009) estimates the level of income for cereals demand ‘saturation’ in around $5000 at purchasing power parity (PPP). This implies very low demand growth in cereals after 2030 for human use and a potentially larger increase in the demand for cereals for energy use.

Overall, these growth projections are very optimistic as they imply income convergence from low- and middle-income countries catching up with developed countries in a similar way to the experience in the last decade. Similarly, FAO (2006) projections use an even more optimistic scenario for income *per capita* growth for the period 2030–2050, largely driven by the slowdown in population growth.

#### (iii) Income distribution projections

Estimates of income inequality and its patterns over time are available for single countries and for the entire world (World Bank 2006; Anand & Segal 2008). Nevertheless, there is lack of agreement regarding the direction of the change in income inequality experienced by the world and by each single country, with some authors arguing that globalization is increasing inequality (e.g. Milanovic 2005) and other authors arguing exactly the opposite (e.g. Sala-i-Martin 2006).

There is, however, a general consensus on two facts, which have important implications for the analysis and the prediction of food demand patterns in the world. First, world income inequality is very high (Anand & Segal 2008). Estimates of the Gini coefficient^5^ for the world income distribution are around and above 0.7, a level of inequality that cannot be found within any single country of the world, not even the most unequal. Second, when income inequality among citizens in the world is decomposed into between- and within-countries inequality, it is found that most of global income inequality (between 80 and 90%) is between-country inequality (Anand & Segal 2008). This implies that shifts in the income distribution of single countries have no significant impact on the world income distribution, unless they occur in countries that are very populous or rich like China, India and the United States. For this reason, it is likely that the dynamic patterns of world food demand will be driven by the patterns of convergence or divergence of the world economies, rather than by changes in the distribution of income within each country.

Bussolo *et al*. (2008*a*) have produced the latest projections on income distribution using a computable general equilibrium (CGE) model with a household module that simulates income distributions (see brief model description below). The authors find that while more income convergence across countries is expected and cross-country inequality is expected to decrease, within-country inequality is going to increase in most of the developing world.

### (b) Existing models for food demand

There are several models used to forecast the future of food demand. Generally, and owing to their main objective—to forecast production, consumption and trade of specific food commodities—these models tend to be partial equilibrium. Table 2 summarizes the existing main models.

**Table 2.** Main food demand models. Source: authors' own elaboration from literature.

model	description
*World Food Model*	
FAO (Alexandratos 2009).	multi-country partial equilibrium model with 52 separate commodities in each country. Simulations are based on assumptions of mean growth of *per capita* income and population. In this model, food for direct human consumption is projected in *per capita* terms using the base year data for this variable. In addition, biofuels enter as demand of food for other uses. Income demand elasticities are constant, and taken from FAO estimates, and complemented with the SWOPSIM model of the United States Department of Agriculture and the MTM model of OECD.
*The World Grains Model*	
The World Bank (Mitchell *et al*. 1997).	non-spatial, partial-equilibrium trade model used to forecast commodity projections. It is also based on constant income demand elasticities.
*The IMPACT model*	
The International Model for Policy Analysis of Agricultural Commodities and Trade (IMPACT); International Food Policy Research Institute.	multi-country/region model where markets are linked through trade. It uses estimated income demand elasticities and covers 36 countries and regions, and 16 commodities, including all cereals, soya beans, roots and tubers, meats, milk, eggs, oils, oilcakes, and meals. Demand is a function of prices, income, and population growth. The IMPACT income demand parameters are based on average aggregate income elasticities for each country, given the income level and distribution of population between urban and rural areas as they evolve over the projection.
*FAPRI model*	
Food and Agricultural Policy Research Institute.	multi-market, partial-equilibrium model of world agriculture, food and biofuel markets. It is based on estimated constant elasticities from the FAPRI elasticity database, and the simulations are based on exogenous changes on growth, population and exchange rates.
*SWOPSIM Model*	
The Static World Policy Simulation; US Department for Agriculture.	comparative statics, multi-product, multi-region partial equilibrium with 20 agricultural commodities. Income demand elasticities are based on estimated elasticities from a large number of sources at the SWOPSIM database.
*Ministerial Trade Mandate* (*MTM*) *Model* OECD	comparative–static model with several country or regional sub-models aimed at analysing policy changes in the medium term. In this model, also exogenous constant elasticities are imposed.
*other models*	*description*
*GIDD model*	
The Global Income Distribution Dynamics (GIDD) model; (Bussolo *et al*. 2008*b*).	model that links simulations from a CGE model to household surveys in order to generate changes and predictions on income distribution. While it has the advantage of considering income dynamics, the demand side of the CGE model is based on a single representative household in each country that maximizes an extended linear expenditure system (ELES). The system captures various substitution possibilities across commodities and shifts in demand towards commodities with higher income elasticities over time. However, changes on income distribution do not feed back to shaping aggregate demand elasticities and the potential bias from aggregation, in addition to linear Engel curves, still remain.
*ENVISAGE model*	
The Environmental Impact and Sustainability Applied General Equilibrium Model (van der Mensbrugghe 2008).	dynamic general equilibrium model calibrated using the GTAP database with 2004 as base year with a carbon emission database. The model can run with the 113 countries/regions and 57 commodity groups of the GTAP database. There is a single representative household that consumes goods and services and saves, and the model is designed with several different consumer demand specifications including the CDE (see Hertel 1997), the LES/ELES (see van der Mensbrugghe 2008) and the AIDADS (see Rimmer & Powell 1996).

Clearly, most of these models fail to incorporate the desired properties listed in the previous section. An initial important limitation of these models is the neglect of general equilibrium interactions with other non-agricultural sectors. More important, however, is the fact that most of these models tend to use simple log demand systems with constant income elasticities (see Islam 1995, for a comparison), which imply no Engel flexibility, especially relevant when considering long-term forecasts. Finally, none of the models disaggregate demand by household type, therefore with risks of aggregation biases if income growth is not distributional-neutral.

### (c) Food demand projections

Several studies have modelled the future of food demand and supply in agriculture. A common conclusion of these studies is that while further pressure on food demand is likely to emerge in coming years, food demand is expected to slow down in future decades aided by low population growth and food ‘saturation’ in emerging markets.

Rosegrant *et al*. (2001) use the IFPRI–IMPACT model in order to analyse the future of agriculture in 2020. The authors suggest that important changes are impacting food demand related to urbanization, rising incomes and lower population growth, particularly in Asia. Demand growth on grains will decline over time. On the other hand, demand for meat will increase in developing countries, while remaining constant in developed countries, increasing pressure on cereals for animal feed. In general, demand growth in developing countries will be higher, increasing the importance of these countries in global food markets.

The main long-term forecasts are produced by the FAO (2006), revised by Alexandratos (2009). According to these forecasts, there is still scope for future demand growth, although almost zero population growth is expected at the global level. This is explained by the fact that most projected population growth will occur in countries with very low consumption levels, mainly in Sub-Saharan Africa. Despite this expected positive demand growth, growth rates are expected to decrease in the future owing to lower population growth and the attainment of medium–high levels of *per capita* consumption in some emerging markets, especially China, which has experienced very high demand growth in the past. While declining growth rates of food for human consumption are expected, these could be compensated by the additional demand arising from biofuels. In addition, meat consumption is also expected to experience some deceleration following increases in *per capita* consumption in China and Brazil, while dairy product demand is expected to continue growing, especially in developing countries. Thus, the main message of the FAO projections is a deceleration in food demand growth rates, especially in the long term.

A key element of the simulation outcomes is the pace of transition towards lower income demand elasticities as income grows and the question becomes how fast this transition will occur. This is especially important, if we consider the recent link between energy and food markets through biofuels, and the recent pressure on cereals for animal feed from increasing meat demand. The potential stress arising from these factors implies that the ability to feed humans will depend on whether the rates of growth of demand for food for human uses are really decreasing at the rates predicted by these studies.

As we have shown during this study, changes in income distribution are crucial to determine food demand growth. Unfortunately, existing projections use models that depart substantially from the optimal demand properties suggested in the previous section. The demand systems used do not have enough Engel flexibility to accurately predict decreasing marginal budget shares. In addition, feedback and general equilibrium effects may be large, especially considering the increasing link between food and energy markets and the potential impact of climate change shocks and policies. Given the uncertainty around income distribution projections in coming decades, existing projections should also consider different scenarios with different distribution dynamics.

Since most inequality in the world is between-country rather than within-country, large changes in income distribution within countries are unlikely to produce large shifts in food demand. Unless the income distribution of populous and rich countries like China, India and the United States change dramatically, little effect can be expected on global food demand from this source alone. The important policy question for demand projections becomes how important would food demand projection errors be if the world would move towards no convergence in the rates of economic growth among countries.

## 5. Changes in income distribution and the future of food demand. how important? an illustration

This section tries to illustrate with a simple example, the size of potential changes in food demand arising from different income distribution scenarios. We perform two simple simulations of the effect of changes in income distribution on food demand within a country and between countries in the world. The simulation is performed using Engel curves estimated by semi-logarithmic functions and by assuming a lognormal distribution of income within countries and in the world. These assumptions are oversimplifying the complex relationship between income distribution and food demand, and other types of simulation are possible. The purpose of the exercise is to provide an initial rough approximation to the size of these effects both within and between countries.

### (a) Within-country inequality and food demand

**Figure 5.**
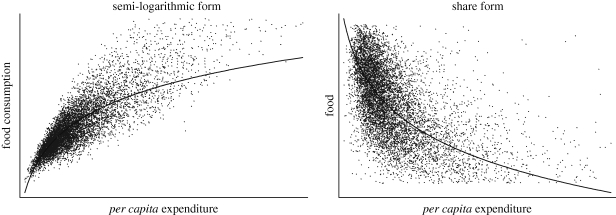
Engel curves in Andhra Pradesh. Source: calculated from NSSO (2006) data of 2005.

In order to provide an example of the effect of changes in income distribution on food demand within an economy, we perform a simulation using data from a sample of households in Andhra Pradesh (India) conducted in 2005 by the NSSO (2006). We estimate the food Engel curve using the semilogarithmic and the food share forms. The fitted values are shown in figure 5. The estimate income elasticity is very high (0.72).

A simple simulation can be performed assuming that the distribution of income is lognormal and exploiting the following property of the lognormal distribution (Prais & Houthakker 1971). The arithmetic mean (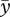) of the lognormal distribution is related to the geometric mean (y*) by the following relationship: 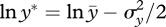, where *σ*_*y*_^2^ is the variance in the distribution of the logarithm of income. Substituting this expression for the mean of logarithm income in equation (2.3), this can be re-written as:
5.1
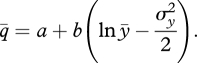



The average food consumption is now a function of both average income and of the variance of income. Mean preserving changes in inequality can be simulated by changing the values of the variance of income. In order to represent the changes in the income distribution with an index, which is more familiar to the readers, and for which data are readily available, we use another property of the lognormal distribution. If the distribution of income is lognormal, the Gini coefficient can be derived from the variance of the lognormal distribution in the following way (Cowell 2008):
5.2
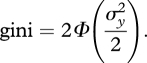



**Figure 6.**
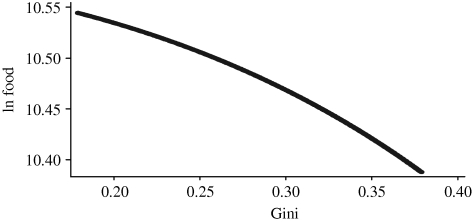
Food consumption and income inequality in Andhra Pradesh. Source: calculated from NSSO (2006) data of 2005.

The initial value of the Gini coefficient is relatively low (0.28). We simulated changes in the Gini of two decimal points above and below the initial value and calculated the corresponding food consumption. The results are displayed in figure 6.

The elasticity of food consumption to the Gini coefficient estimated from this curve is approximately −0.2, indicating that an increase by 10 per cent in the Gini, a change not far from those normally observed within one or a few years, would reduce *per capita* food consumption by about 2 per cent.

This simulation is valid under the assumption that the shape of the Engel curve does not vary over time, which is equivalent to assuming that changes in income levels over time for the same household are mirroring income changes across different households at a single point in time. In other words, food consumption of households with different income levels is used to predict food consumption by the same household when its income increases. Another way to express this is that the shape of the Engel curve should not vary over time. There are a number of reasons, however, that suggest that the shape of the curve might vary over time. Firstly, households may stick to their diets for some time after income changes rather than quickly adopting the diets of richer households. In this case, the change in food consumption over time would be smaller than the change in consumption over the cross section of households, and the Engel curve would be steeper. Secondly, consumer preferences, which determine consumption with income and prices, may change, and as a result alter the shape of the Engel curve. One obvious case is the change in family composition or size over time, because people of different age have different consumption needs. Children in particular tend to consume proportionally more food than adults in developing countries. In this case, the change in food consumption over time, following, for example, a decrease in average family size, would be larger than the change in consumption over the cross section of households, and the Engel curve would be flatter. Finally, new goods can be introduced over time and food luxury items may appear on the market, which attract new consumers. The introduction of these items might shift upwards the right section of the Engel curve putting into question the existence of a ‘saturation’ point. All these possible effects suggest that the invariance of the shape of the Engel curve over time should be tested with the survey data rather than assumed. If Engel curves can be estimated from several cross-sectional surveys that are years apart from each other, then parametric and non-parametric tests are available to assess the stability of the curves over time.

We also simulated the pattern of *per capita* food consumption over the next 40 years. To do so we assumed a rate of *per capita* income growth of 4.1 per cent per year.^6^ As *per capita* income increases over time, *per capita* food consumption moves along the food Engel curve. However, if the incomes of the poor and the rich grow at different rates and the distribution of income varies, then the patters of food consumption deviate from the Engel curve. We estimated a very large change in the Gini coefficient by ±20 per cent^7^ over a 40 year period, and calculated the levels of *per capita* food consumption for each year.

**Figure 7.**
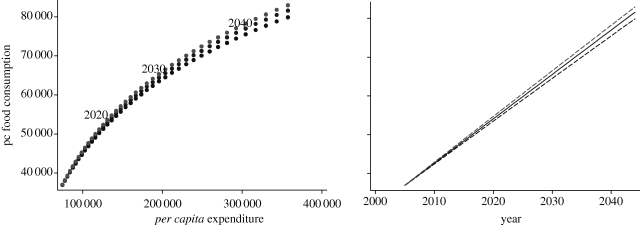
Projected food consumption in Andhra Pradesh over the next 40 years. Source: calculated from NSSO (2006) data of 2005.

The results of these simulations are shown in figure 7. A more egalitarian distribution has the effect of increasing *per capita* food consumption, while a less egalitarian distribution produces the opposite outcome. The chart on the left of figure 7 shows the deviations from the Engel curve, while the chart on the right shows the levels of *per capita* food consumption over time. Under the assumptions made in the simulation, an increase in inequality by 20 per cent over 40 years will produce a reduction in *per capita* food demand by about 2 per cent. Conversely, a reduction in the Gini coefficient by 20 per cent would result in 40 years in an increase in *per capita* food consumption by about 1.6 per cent. Clearly, the impact of changes of income distribution, even when considering very large changes, on *per capita* food demand in this case is small, since inequality in Andhra Pradesh is not high.

**Figure 8.**
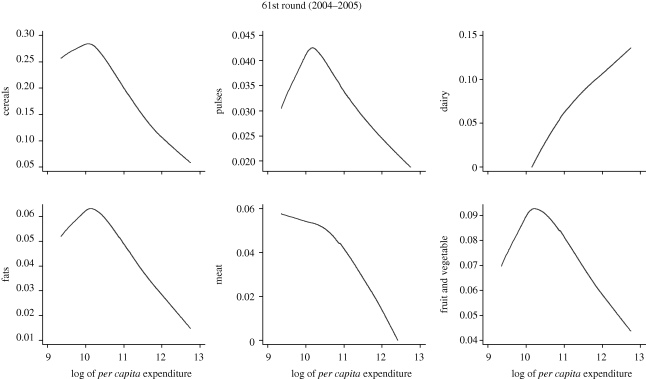
Engel curves for six different categories in share form. Source: calculated from NSSO (2006) data.

Regarding food composition, the effects of changes in income distribution on specific food items depend on the shape of the Engel curve for that particular item. Figure 8 displays Engel curves in share form for six broad food categories (cereals, pulses, dairy, fats, meat, and fruit and vegetable) for the sample of household in Andhra Pradesh. Expenditure is expressed as a share of total expenditure. In this way, a concave down Engel curve implies a falling share, while a concave up form implies an increasing share. The curves were calculated employing semi-parametric methods in order to let the data define the shape of the curve rather than imposing any specific functional form (Yatchew 2003).

The Engel curve for cereals has a concave down shape for most of the expenditure range, but is concave up for very poor households. Only the Engel curves for dairy and meat show a clear pattern. Dairy is consumed more than proportionally as expenditure increases, while the share of expenditure in meat consistently decreases as expenditure increases.^8^ Changes in income distribution for these food items are clearly predictable. An increase in inequality increases the consumption of dairy products and reduces the consumption of meat. The effects of changes in income distribution on the consumption of other food items are much less predictable because the Engel curves in share form have an inverted U shape. Whether consumption increases or decreases depends on the nature of the transfer taking place. For example, while as a general rule an increase in inequality reduces consumption of cereals, if the increase in inequality is circumscribed among the very poor (the rising section of the cereals Engel curve in figure 8), cereals consumption increases.

### (b) Between-country inequality and food demand

**Figure 9.**
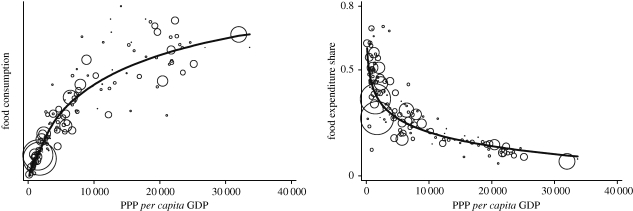
World food Engel curves. Source: calculated from the International Comparison Programme data.

Since data are available on food consumption and *per capita* gross domestic product (GDP) for most countries of the world, it is tempting to proceed to the estimation of a world food Engel curve and to perform a world-scale simulation of a change in the world income distribution. Figure 9 shows the estimation of semilogarithmic and share form food Engel curves for most countries in the world using the data of the International Comparison Programme of the World Bank. Circles represent countries, and their size is proportional to the population of each country. The large circle of the United States on the right, and of India and China on the left, are clearly visible. The world income elasticity of food consumption estimated by these curves is 0.48.

The simulation of the effect of changes in the world income distribution on world food demand can be performed in the same way as within a single country. However, before carrying out the simulations, the concept of world income inequality needs to be properly defined. There are three main concepts of income inequality in the world (Milanovic 2005). The first concept, *international inequality*, is inequality between states, where each country is represented by one single observation. This concept is clearly inappropriate for the present analysis because it is not representative of consumption behaviour in the world. The second concept, *global inequality*, is inequality among world citizens as could be obtained by running a random income survey over the entire world. This concept represents the true income inequality in the world but data for its calculation are not readily available. The third concept, *between-country inequality*, is inequality between countries, where each country is represented by the number of its citizens. In other words, this concept measures inequality among individuals, but each individual is assigned the average income of her country of residence. This is the concept used in estimating the charts of figure 8. Between-country inequality ignores within-country inequality and, as any form of income averaging by aggregation, it underestimates the true global world inequality. It is estimated, however, that between-country inequality captures around 80 per cent of global inequality (Anand & Segal 2008), and it represents therefore a good approximation of global inequality. In our data, the estimated world Gini coefficient is 0.56, which compares relatively well to estimates of 0.7 often found for global income inequality.

The world income distribution is assumed to be normal in the logarithms as it was the case of the within-country distribution. There is not strong support in the data for this particular parametric form as the true distribution of income, including within-country inequality, is not known, but it is an approximation often used by studies investigating world inequality (e.g. Chotikapanich *et al*. 1997; Dowrick & Akmal 2005).

**Figure 10.**
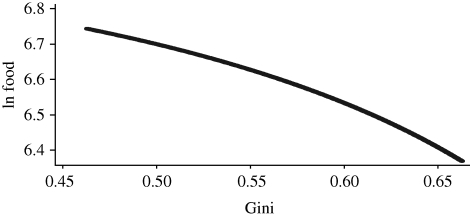
Food consumption and income inequality in the world. Source: calculated from the International Comparison Programme data.

Figure 10 shows the relationship between the world average food consumption and the world Gini coefficient. The elasticity of food consumption to the Gini coefficient estimated from this curve is −1, indicating that a reduction by 1 per cent in the world Gini would increase *per capita* food consumption by the same percentage amount. This elasticity is much larger than the one observed within-country, presumably a consequence of the different curvature of the world Engel curve and of the world income distribution compared with the national ones. Year-to-year estimates of world Gini are not easily available because of difficulties in obtaining the required data. Sala-i-Martin (2006), for example, estimated a reduction in global income inequality by 0.8 per cent over 1992 and 1993. According to our estimates, a 0.8 per cent increase in *per capita* global food demand should have occurred over the same years only on account of a change in the world distribution of income.

**Figure 11.**
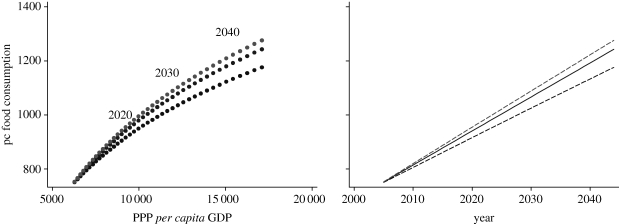
Projected world *per capita* food consumption over the next 40 years. Source: calculated from the International Comparison Programme data.

We also repeated the simulation performed for Andhra Pradesh for the whole world, assuming a *per capita* income growth of 2.6 per cent per year. We predicted trends in the world distribution of income using historical series of the Gini coefficient calculated by Sala-i-Martin (2006) and by Milanovic (2009). The trends are extrapolations over time of the Gini of global income distribution calculated from an ‘optimistic’ point of view (Sala-I-Martin) and a ‘pessimistic’ point of view (Milanovic), respectively. Theoretically, the two views are supported by diverging hypotheses regarding convergence or divergence of world economies. The results are shown in figure 11. The effect of changes in the income distribution is considerable. Under the assumptions made, an increase in Gini inequality by 8 per cent over 40 years will produce a reduction in *per capita* food demand by about 5.4 per cent, while a reduction in the Gini coefficient by 5 per cent would result in 40 years in an increase in *per capita* food consumption by about 2.7 per cent compared with the income distribution-neutral growth case. These effects, however, are not large if compared with the effects of increases in *per capita* income. For example, the increase in consumption of food following *per capita* income growth alone, independently of changes in income distribution, over the same period of 40 years is nearly 50 per cent. Given the estimated expenditure elasticity of world food consumption (0.47), the increase in food consumption obtained under the most optimistic prediction of a 5 per cent fall in the Gini over 40 years might be obtained in just slightly over 2 years of *per capita* income growth at the current levels.

The much larger effect of income distribution on food demand between countries than within countries is explained by the wider differences in demand elasticities between countries in the world compared with the differences between individuals within countries. This illustration underlines the point that shifts in food demand deriving from changes in the income distribution are to be expected to originate from the patterns of convergence or divergence between world economies rather than from the trends in inequality within each country. However, it should also be emphasized that predictions of future world income distribution were obtained by extrapolating calculated series of Gini coefficients. Without some strong theoretical assumptions regarding convergence or divergence of world economies, it is difficult to interpret these extrapolations as ‘trends’. These predictions are, to some extent, only hypothetical: the increasing inequality trend might be reversed and vice versa.

## 6. Conclusions and directions for future research

This paper has surveyed the relationship between income distribution and food demand. The cornerstone of this relationship is Engel's law, which establishes that food budget shares decrease as income grows. One implication of Engel's law is that any accurate prediction of future food demand needs to deal with two main issues when considering income distribution: aggregation across households and Engel flexibility. The former refers to the fact that considering only mean income growth to determine aggregate food demand can be misleading when income growth is different across income groups with different income elasticities. The latter refers to the fact that as income grows in time, households and countries move towards decreasing food budget shares.

The review of the literature on demand systems suggests that only rank three demand systems produce nonlinear food budget shares on income, and that accurate aggregation requires moving away from representative consumer models to aggregation across different types of households. Furthermore, models should integrate general equilibrium effects related to feedback from agriculture to income distribution and the link between energy and food markets via biofuels.

The review of existing models for forecasting food demand found that most of these models do not comply with any of the properties suggested above. As a result, the accuracy for existing food demand projections is highly conditional on the assumptions of within-country distributional-neutral growth and continuous reduction in cross-country inequality. A less plausible scenario of growth divergence would slow down food demand growth in the short term, while at the same time making food demand growth more persistent in time. These potentially different demand growth outcomes in the short, medium and long term are important when considering additional stresses on demand arising from high energy prices and potential climate change shocks and policies.

A key question that arises from the paper is how deviations from income distribution projections may impact the accuracy of food demand projections. In order to answer this question, the paper carries out some simple simulations. Two main results emerge from the simulations. First, the largest potential impact on world food demand comes from changes in between-country inequality, rather than within-countries inequality. Second, considering two different income distribution scenarios, one optimistic and one pessimistic, world food demand in 2050 would be 2.7 per cent higher and 5.4 per cent lower than the one under distributional-neutral growth. Thus, income distribution changes have an impact on food demand projections.

Clearly, more research is needed in this area. To begin with more empirical work is required to analyse the extent of the demand bias arising from not considering potential income distribution changes. Secondly, more realistic models integrating more sophisticated demand systems with higher Engel flexibility are required to forecast the future of food demand. Thirdly, changes in demographic structure of the countries should be taken into account by both estimation and modelling exercises. Finally, general equilibrium models for disaggregated food commodities able to include different types of households, linking food and energy markets, and capable of feeding back into the model income distribution changes are desirable.

## Endnotes

1 In economics the responsiveness of demand to income changes is called income demand elasticity. Engel's Law implies that different households have different food income demand elasticities according to their levels of income, and as income grows within households, income demand elasticities tend to decrease over time.

2 See the electronic supplementary material, appendix for a more detailed description on household composition and aggregation.

3 A more detailed review of the main demand systems used in the empirical and modelling literature can be found in the electronic supplementary material, appendix.

4 Cranfield *et al*. (2003) suggest that individual aggregations is possible under linear expenditure system, quadratic expenditure system, AIDS and QUAIDS, but not under AIDADS.

5 The Gini coefficient was developed in 1912 by Italian statistician Corrado Gini. It is a statistical dispersion that is mainly used as a measure of inequality of income and wealth.

6 This is the rate predicted by FAO (2006).

7 Within-country Gini coefficients tend to be very stable over time. As a result a 20 per cent change is a very large and unlikely change in income distribution.

8 Indian households are largely vegetarian and increasingly so, as the poorer section of society adopt the consumption habits of middle and upper classes.
